# Impact of the gut-lung axis on tuberculosis susceptibility and progression

**DOI:** 10.3389/fmicb.2023.1209932

**Published:** 2023-07-06

**Authors:** Aditya Enjeti, Harindra Darshana Sathkumara, Andreas Kupz

**Affiliations:** ^1^College of Medicine and Dentistry, James Cook University, Townsville, QLD, Australia; ^2^Centre for Molecular Therapeutics, Australian Institute of Tropical Health and Medicine, James Cook University, Cairns, QLD, Australia

**Keywords:** tuberculosis, microbiome, gut-lung-axis, immunity, dysbiosis, probiotics

## Abstract

Tuberculosis (TB) has remained at the forefront of the global infectious disease burden for centuries. Concerted global efforts to eliminate TB have been hindered by the complexity of *Mycobacterium tuberculosis* (*Mtb*), the emergence of antibiotic resistant *Mtb* strains and the recent impact of the ongoing pandemic of coronavirus disease 2019 (COVID19). Examination of the immunomodulatory role of gastrointestinal microbiota presents a new direction for TB research. The gut microbiome is well-established as a critical modulator of early immune development and inflammatory responses in humans. Recent studies in animal models have further substantiated the existence of the ‘gut-lung axis’, where distal gastrointestinal commensals modulate lung immune function. This gut microbiome-lung immune crosstalk is postulated to have an important correlation with the pathophysiology of TB. Further evaluation of this gut immunomodulation in TB may provide a novel avenue for the exploration of therapeutic targets. This mini-review assesses the proposed mechanisms by which the gut-lung axis impacts TB susceptibility and progression. It also examines the impact of current anti-TB therapy on the gut microbiome and the effects of gut dysbiosis on treatment outcomes. Finally, it investigates new therapeutic targets, particularly the use of probiotics in treatment of antibiotic resistant TB and informs future developments in the field.

## Introduction

### Tuberculosis

Tuberculosis (TB) remains at the forefront of the global disease burden with over 10 million new cases and 1.6 million deaths in 2021 (World Health Organization, 2023). Currently, it is the second highest infectious cause of death after COVID19 and has a disproportionate socioeconomic impact on low to middle income countries through catastrophic healthcare costs for individuals (World Health Organization, 2023). Current global initiatives to target TB are centered around addressing social determinants, early case identification and provision of effective antibiotics in the framework of universal healthcare (World Health Organization, 2022). Concerted global efforts led by the WHO through the END TB strategy and sustainable development goals have led to a gradual reduction in case incidence over the past decade (World Health Organization, 2022). However, the COVID19 pandemic is estimated to have reversed progress by 12 years through its disruption to case detection and treatment access, with consequent increases in mortality and morbidity (Wingfield et al., 2021; World Health Organization, 2022). Additionally, the emergence of drug resistant mycobacteria strains has rendered many first line antibiotic regimens ineffective, driving the resurgence of TB in low and middle income countries (Tiberi et al., 2022). Novel vaccine development remains hindered by an incomplete understanding of *Mycobacterium tuberculosis* (*Mtb*) pathogenesis and spreading of multidrug resistance threatens to further negate progress (McShane, 2019; Stephanie et al., 2021).

### The gut microbiome

A novel avenue for TB treatment and management is modulation of the gut-lung axis – the bidirectional relationship between the composition and metabolism of the gastrointestinal microbiome and regulation of lung immune responses recently evidenced in animal studies (Mori et al., 2021). The gut microbiome has long been recognized for its multifaceted role in health through detoxification, protection against pathogens, regulation of metabolism and modulation of the immune system (Wu and Wu, 2012; Zheng et al., 2020). Germ free (GF) animal models have defective lymphoid tissue, increased likelihood of Th1/Th2 imbalances as well as reduced intraepithelial lymphocytes, IgA antibodies and Th17 immunoregulatory cells (Zheng et al., 2020). Microbiome development is influenced by a range of factors including maternal diet, infections, probiotic use, genetics, geography, delivery method, gestational age, diet, and antibiotic use (Vandenplas et al., 2020; Li P. et al., 2022; Wernroth et al., 2022). The infant microbiome composition is highly dynamic in response to these environmental factors (Li P. et al., 2022; Wernroth et al., 2022), and the diversity generated in this period modulates IgE homeostasis, determining allergic susceptibility (Méndez et al., 2021; Zhu et al., 2021).

Short chain fatty acids (SCFAs), such as butyrate, are produced by the gut microbiota and function as signaling molecules that modulate inflammatory responses, regulate macromolecule metabolism and reduce colorectal cancer risk (He et al., 2020). They modulate pH, regulate mucus production and act as colonic epithelial cell energy source, directly promoting gut integrity (Blaak et al., 2020). Butyrate reduces IL-12 and IFNγ production by inhibiting histone deacetylase (HDAC), mammalian target of rapamycin (mTOR) kinase and nuclear factor kappa B (NF-κB) signaling, preventing skewing to the inflammatory Th1/M1 phenotype (He et al., 2020; Kotlyarov, 2022). Furthermore, butyrate inhibits nitric oxide and LPS-mediated induction of proinflammatory cytokines such as IL-6, IL-12, IL-1β, and TNFα (He et al., 2020; Kotlyarov, 2022). SCFAs also inhibit IL-12 release from dendritic cells (DC), inhibiting antigen specific CD8^+^ T cell activity and increasing infection risk (Nastasi et al., 2017). While predominately acting anti-inflammatory, SCFAs can also produce pro-inflammatory states through G protein-coupled receptors (GPCRs), and promote CD8^+^ T cells memory potential (Bachem et al., 2019; He et al., 2020). High butyrate or propionate levels in children are also protective against the development of atopy (Roduit et al., 2019). Thus, microbiome composition and SCFA production has a significant impact on immune development and dysfunction with lasting implications for health in adult life (Zheng et al., 2020).

### Gut-lung axis

Emerging evidence supports the role of gut microbiota in modulating immunity and inflammation at distal sites such as the lungs (Osei Sekyere et al., 2020). Changes in microbiota metabolites or composition correlate to defective immune responses in many respiratory diseases (Comberiati et al., 2021). Animal models demonstrated more severe *Escherichia coli* pneumonia in mice with gut commensal depletion due to decreased alveolar macrophages (AM) activity via reduced toll-like receptor (TLR) signaling, NF-κB DNA-binding activity, TNFα, CXCR2 and ICAM expression on intestinal mucosa (Chen et al., 2011). Similarly, a murine model of *Streptococcus pneumoniae* infection found that antibiotic treated mice given fecal suspensions by oral gavage showed enhanced AM function (Schuijt et al., 2016), however antibiotic effects and oral gavage use may have confounded results (Budden et al., 2017). *Clostridium* spp. may be an important regulator of allergic asthma through the induction of IL10^+^ CTLA4^+^ colonic T regulatory cells (Tregs) (Di Gangi et al., 2020). Animal models demonstrate antibiotics specific to *Clostridium spp*., such as vancomycin, reduce CD4^+^CD25^+^ Tregs (Di Gangi et al., 2020). Recent evidence suggests that *Helicobacter pylori* infection is inversely correlated with asthma severity (Chen et al., 2011; Lim et al., 2016; Kato et al., 2017; Tsigalou et al., 2019), however this is contradicted by other studies (Wang et al., 2012, 2013, 2017; Molina-Infante et al., 2018), suggesting its role remains unclear.

**Figure 1:**
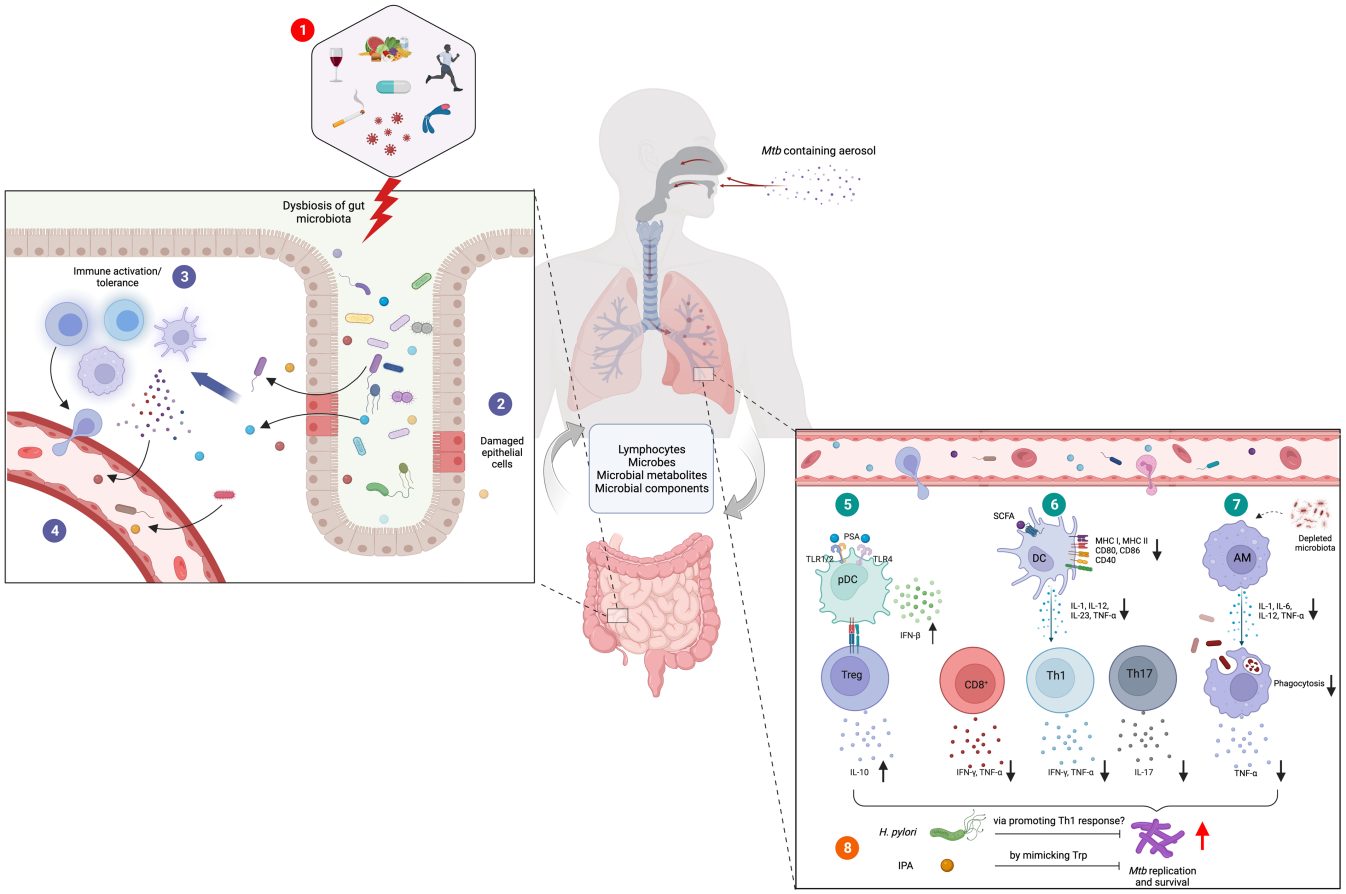
Role of gut-lung axis in *Mtb* infections. **(1)** Diet, alcohol, smoking, infectious diseases, lifestyle habits, antibiotics and genetic conditions are among the major causes of gut dysbiosis. **(2)** Altered gut microbiota cause damages to the gut epithelial layer facilitating the translocation of microbes, microbial components, and metabolites into the tissue **(3)** followed by immune activation. **(4)** Some of these activated immune cells, immune molecules, microbes, and their metabolites migrate to distal sites through blood circulation. **(5)** In the lung tissue, bacterial components such as PSA induce the secretion of IFNβ from plasmacytoid DC (pDCs) and activation and expansion of Tregs. **(6)** Microbial metabolites such as SCFA downregulate the expression and secretion of co-stimulatory molecules and cytokines in DCs. This results in defective Th1 and Th2 responses and diminished antigen specific CD8^+^ T cell activation. **(7)** Depleted gut microbiota hinders the phagocytic capacity of AMs supporting the survival and growth of *Mtb*. Certain organisms and their metabolites have demonstrated anti-tubercular activities. **(8)** *H. pylori* and IPA are believed to restrict *Mtb* survival by promoting pro-inflammatory Th1 response and mimicking Trp, respectively. Figure was created with Biorender.com.

Polysaccharide A (PSA), produced by *Bacteroides fragilis*, suppresses adverse inflammatory responses, inhibiting asthma pathogenesis (Johnson et al., 2018). PSA signals through the TLR2/TLR1 heterodimer to activate multiple signaling pathways that promote immune tolerance such as activating IL-10 producing Tregs (Figure 1; Erturk-Hasdemir et al., 2019). Furthermore, PSA also induces dose dependent interferon beta (IFNβ) expression by colonic lamina propria DCs through TLR4 activation which has been shown to be protective in infection with vesicular stomatitis virus or influenza A virus (Stefan et al., 2020; Wirusanti et al., 2022). This supports the hypothesis that microbiota critically modulate homeostatic type 1 IFN expression essential for a rapid antiviral response and effective viral clearance (Van Winkle et al., 2022; Wirusanti et al., 2022).

Gut dysbiosis results in systemic inflammation and bacterial translocation, eventuating in lung dysbiosis (Donovan et al., 2020). This process may activate the inflammasome NOD-like receptor protein 3 (NLRP3) triggering sterile inflammation and neutrophil recruitment that could cause gut epithelial damage and increased permeability (Donovan et al., 2020; Liu Q. et al., 2021). Dysbiosis is also strongly correlated with inflammatory bowel disease (IBD), with 60% of IBD patients also having subclinical lung disease (Raftery et al., 2020). SCFAs regulate lung immune tone by binding to free fatty acid receptors (FFAR), a classic GPCR found on AMs, causing basal IL-1β expression and modulating type 1 IFN responses to respiratory syncytial virus (RSV) infections (Liu Q. et al., 2021). Additionally, patients with severe chronic obstructive pulmonary disease (COPD) were found to have lower SCFAs levels, possibly due to their role in epithelial barrier integrity via increased ZO-1 dense contact proteins expression (Kotlyarov, 2022). Microbiota have also shown to be protective against *Streptococcus pneumoniae* infection through enhancement of primary AM phagocytosis and responsiveness to pathogenic associated molecular patterns (PAMPs) (Schuijt et al., 2016).

## The gut-lung axis in *Mycobacterium tuberculosis* infection

Millions of individuals acquire a latent or active TB infection annually without obvious immune deficiency, indicating the existence of previously unidentified risk factors (Namasivayam et al., 2018). Alterations in the gut-lung axis is hypothesized to be a contributing factor in *Mtb* infection pathogenesis and its clinical presentation (Comberiati et al., 2021). Early GF models initially suggested no difference in *Mtb* tissue load between GF and conventional mice (Suter and Kirsanow, 1962; Huempfner and Deuschle, 1966), however recent developments in the understanding of gut microbiome-mediated immunomodulation has renewed interest in the area. Human immunodeficiency virus (HIV) infection, malnutrition, diabetes, alcohol, smoking and air pollution which are important risk factors for TB have all also been shown to cause changes in the gut microbiome (Naidoo et al., 2019). These factors and others can cause gut dysbiosis (Figure 1), resulting in alterations in the microbiota’s biosynthetic pathways, changes in the lung microbiome and downstream immunomodulatory effects (Shah et al., 2021). This results in reduced resistance to colonization by external pathogens, escape of a contained pathogen or loss of benign lung commensals, leading to lung disease (Naidoo et al., 2019).

C3HeB/FeJ mice fed with a high fat diet showed proinflammatory responses that increased the risk for developing active TB and impaired the immune protection from BCG vaccination in obese mice (Arias et al., 2019). The authors hypothesized that this was due to a reduction in the *Firmicutes/Bacteroidetes* phyla ratio and decreased *Porphyromonadaceae* family abundance in the gut microbiota (Arias et al., 2019). Additionally, increases in the genera associated with dysbiosis such as *Alistipes*, *Parasuterella*, *Mucispirillum*, and *Akkermansia* were observed (Arias et al., 2019). However, an increased *Firmicutes/Bacteroidetes* ratio was found in a murine model of type 2 diabetes (T2D) generated through the administration of an energy dense diet (Sathkumara et al., 2021). The study also suggested that these changes in the gut microbiome may increase susceptibility to TB through alterations in SCFA metabolism (Sathkumara et al., 2021). Additionally, rhesus macaque monkeys that developed severe disease from *Mtb* infection had distinct intestinal microbiota compared to those with less severe disease (Namasivayam et al., 2019). In particular, animals with more severe disease had enriched *Lachonospiraceae* and *Clostridiaceae* and depleted *Streptococcaceae* bacterial families (Namasivayam et al., 2019). These findings, support the importance of factors that impact gut dysbiosis due to crosstalk on the gut-lung axis and implications for TB pathogenesis and susceptibility.

Aerosol *Mtb* infection in mice can lead to rapid changes in intestinal bacterial composition, particularly in the orders *Closteridiales* and *Bacteroidetales* (Winglee et al., 2014). A low dose *Mtb* aerosol challenge caused rapid intestinal dysbiosis and loss of diversity in both diabetic and nondiabetic mice in a murine model of T2D (Sathkumara et al., 2021). A similar study of *Mtb* infection in a murine model found similar trends but these were not significant over the duration of the study (Namasivayam et al., 2017). Patients with a *Mtb* respiratory infection have been shown to have reduced gut microbiome diversity compared to healthy controls, evidencing the role of the gut-lung axis in susceptibility to TB (Hu et al., 2019; Comberiati et al., 2021). Pulmonary *Mtb* infection was also shown to decrease the α diversity of the gut microbiome, particularly through alterations in the populations of the genus *Bacteroides* in recent human studies (Hu et al., 2019; Wang Y. et al., 2022; Wang S. et al., 2022). One cross-sectional study contradicted these results by finding no significant alterations in gut microbiota in latent TB patients, but the same patients were not sampled before and after infection (Wipperman et al., 2017; Naidoo et al., 2019).

Gut bacteria from the phylum *Bacteroidetes*, which include many beneficial commensals, were also found to be reduced in patients with recurrent TB (Table 1; Luo et al., 2017). Species from the genera *Lachnospira* and *Roseburia* (phylum *Firmicutes*) were also depleted in TB patients, with possible negative ramifications for the production of SCFAs and their consequent downstream regulatory effects (Luo et al., 2017). Species from the more pathogenic phyla *Actinobacteria* and *Proteobacteria* including *Escherichia coli* were increased in recurrent TB patients’ feces samples, consistent with previous studies analyzing sputum (Luo et al., 2017). Both the genera *Prevotella* and *Lachnospira* were shown to inversely correlate with recurrent TB, positively correlate with peripheral CD4^+^ cell counts in new cases and decrease in both new and recurrent TB patient groups (Luo et al., 2017). However, in a HIV^+^ positive population on anti-retroviral treatment with high TB incidence, increased oral anaerobes including species of the genus *Prevotella*, were associated with higher levels of pulmonary SCFA that positively correlated with risk of developing active TB (Segal et al., 2017).

**Table 1:** Summary of the gut microbiome changes in patients infected with *Mtb* relative to healthy controls in key human studies.

Study	Population	Increased	Decreased
Hu et al. (2019)	31 healthy controls and 30 newly diagnosed active pulmonary patients	*Coprobacillus* bacterium and *Clostridium bolteae*	*Roseburia*, *Coprococcus* and *Eubacterium* genera
Luo et al. (2017)	19 new tuberculosis patients (NTB), 18 recurrent tuberculosis patients (RTB) and 20 healthy controls.	*Actinobacteria* and *Proteobacteria* phyla in RTB patients	*Firmicutes* (specifically *Lachnospira* and *Roseburia* genera) and *Bacteroidetes* phyla in RTB patients *Coprococcus* and *Roseburia* genera in NTB patients
Li et al. (2019)	18 pediatric pulmonary tuberculosis patients and 18 healthy pediatric controls	*Prevotellaceae* (*Bacteroidetes* phyla) and *Enterococcaceae* (*Firmicutes* phyla) families	*Ruminococcus* and *Faecalibacterium* genera (*Firmicutes* phyla), and *Bifidobacteriaceae* family (*Actinobacteria* phyla)
Maji et al. (2018)	6 tuberculosis patients and 6 healthy controls (blood relative of patient)	*Faecalibacterium, Roseburia*, *Eubacterium* (specifically *E. rectale*), *Butyvibrio* (*Firmicutes* phylum) and Phascolarctobacterium genera	*Prevotella* (*Bacteriodetes* phyla) and *Bifidobacterium* genera (*Actinobacteria* phyla)
Naidoo et al. (2021)	Patients presenting with suspected TB were classified as cases (*n* = 58) or symptomatic controls (*n* = 47), and compared against close contacts of cases (*n* = 73) and close contacts of controls (*n* = 82) from the same household	*Lachnospiraceae* (particularly *Anerostipes* and *Blautia* genera) and *Erysipelotrichaceae* families in cases compared to symptomatic controls	*Bifidobacterium, Roseburia, Dorea* genera compared to close contacts of cases

A case–control study of pulmonary TB in pediatric patients also found significant gut dysbiosis in comparison to healthy controls with enriched *Prevotellaceae* and *Enterococcaceae*, but decreased beneficial *Oscillospiraceae* and *Bifidobacteriaceae* bacterial families (Table 1; Li et al., 2019). The authors speculated that the increased populations of *Prevotella* may induce a proinflammatory cytokine production that worsens TB, though this remains to be mechanistically confirmed (Li et al., 2019). *Faecalibacterium ruminococcaceae* and *Faecalibacterium prausnitzii* species depletion (part of the *Oscillospiraceae* family) may have negative effects through reduced immunomodulatory SCFAs while reductions in the bacterial family *Bifidobacteriacae* have been associated with many other respiratory diseases (Li et al., 2019).

A study examining gut microbiome alterations in TB patients through 16S rRNA gene and whole-genome shotgun sequencing also found significant depletion of *Provetalla* and increased *Bacteroides* intestinal bacterial genera (Table 1; Maji et al., 2018). Considerable increases in butyrate and propionate producing intestinal bacteria in *Faecalibacterium, Roseburia, Eubacterium and Phascolarctobacterium* genera were detected among TB patients (Maji et al., 2018). Pre-treatment TB patients also showed increased butyrate-producing anaerobic species from the bacterial families *Lachnospiraceae* and *Erysipelotrichaeceae* in stool samples compared to healthy controls (Table 1; Naidoo et al., 2021). Butyrate inhibits IFNγ and IL-17A, strongly reducing Th17 proliferation and causing a detrimental dysregulation of the immune response against *Mtb* (Lachmandas et al., 2016; Segal et al., 2017; Naidoo et al., 2021). Additionally, through induction of immunosuppressive Tregs in the gut and consequent IL-10 release, butyrate may suppress critical pro-inflammatory T cell responses in TB patients facilitating immune evasion and chronic infection (Figure 1; Maji et al., 2018). Furthermore, this study showed a positive correlation between species from the *Lachnospiraceae* and *Erysipelotrichaceae* family and interferon regulation, inflammasome activation, cell death signaling and antibacterial activity in TB patients (Naidoo et al., 2021).

In contrast, a small cohort study found that healthy controls have higher levels of SCFA producing bacteria in their gut microbiota, such as the butyrate-producing *Roseburia*, *Coprococcus* and *Eubacterium* genera, compared to TB patients (Table 1; Hu et al., 2019). TB patients had altered activity of microbiome metabolic pathways through decreased production of precursor metabolites and energy, decreased degradation/utilization/assimilation capacities and increased vitamin synthesis (Hu et al., 2019). The study authors found that active TB patients had a unique microbiota signature through strain level single nucleotide polymorphisms and species patterns that differentiates them from healthy controls (Hu et al., 2019).

Colonization by *Helicobacter* spp. has diverse effects on the pathogenesis of TB. Murine models of *Helicobacter hepaticus* show increased inflammation, severe lung pathology, increased *Mtb* burden and worsened mortality and morbidity after aerosol challenge (Arnold et al., 2015; Majlessi et al., 2017). This is postulated to result from increased IL-10 (Cervantes and Hong, 2017), which suppresses macrophage activation and DC function in the early immune response to *Mtb* (Redford et al., 2011). *Helicobacter pylori* in contrast may induce a protective immunomodulatory response that reduces the TB risk in humans (Perry et al., 2010). Individuals with *H. pylori* infections were found to have heightened IFNγ and Th1-like responses to *Mtb* antigens (Figure 1), more likely to maintain latency and less likely to develop active disease (Perry et al., 2010, 2013). However, these results contradict the results of three studies which found no association between *Mtb* and *H. pylori* (Sanaka et al., 1998; Tsang et al., 1998; Torres et al., 2003), and three studies that found that *H. pylori* increased the incidence of *Mtb* infections (Mitchell et al., 1992; Woeltje et al., 1997; Philippou et al., 2003). *H. pylori* may worsen TB through its ability to increase Treg populations, interfere with DC maturation and prevent T lymphocyte maturation (Bustamante-Rengifo et al., 2021). However, the exact relationship between *H. pylori* and *Mtb* remains to be fully elucidated through larger studies with appropriate controls.

## TB, antibiotics, and BCG

Murine models have consistently demonstrated that antibiotics can cause dramatic alterations in the gut microbiome,(Khan et al., 2016; Namasivayam et al., 2017; Dumas et al., 2018) potentially contributing to TB pathogenesis and affecting treatment efficacy. A mouse model of dysbiosis induced by the administration of wide-spectrum antibiotics showed increased colonization of the lungs by *Mtb* and reduced mucosal associated invariant T (MAIT) cells during the first week of infection (Dumas et al., 2018). Inoculation with a gavage of microbiota from non-treated mice reversed these changes and regenerated the MAIT cell population (Dumas et al., 2018). Another study of antibiotic therapy in a murine model of TB also demonstrated significant alterations in the microbiome (Khan et al., 2016). The antibiotic regimen was chosen to specifically not change *Mtb* viability but solely cause gut dysbiosis (Khan et al., 2016). Mice on the antibiotic therapy had higher *Mtb* burden both in the lungs and extra-pulmonary sites (Khan et al., 2016). This likely resulted from the suppression of Th1 immunity and enhancement of Tregs due to the alterations in gut microbiota (Khan et al., 2016). Furthermore, fecal transplants were effective to rebuild the gut microbiota, improve Th1 immunity, inhibit Treg populations and reduce *Mtb* burden (Khan et al., 2016). Similarly, treatment of mice with widely used anti-TB drugs isoniazid and pyrazinamide prior to *Mtb* infection resulted in significant gut dysbiosis and increased *Mtb* burden (Khan et al., 2019). This was associated with impaired AM metabolism and defective bactericidal activity and was reversible with fecal transplantation form untreated animals (Khan et al., 2019). These antibiotics are associated with more selective alterations in the gut microbiome including decreases in the *Closteridia* genera,(Khan et al., 2019) associated with Treg induction (Atarashi et al., 2013).

The use of broad-spectrum antibiotics on *Mtb* infected mice showed that the antibiotics caused significant gut dysbiosis that resulted in the deregulation of approximately 7,592 long noncoding RNA (lncRNA) sequences (Yang et al., 2022). One especially depleted highly conserved lncRNA, temporarily named lncRNA-CGB (commensal gut bacteria associated lncRNA), was especially downregulated in both mouse models and humans with active TB, correlating with poor outcomes in *Mtb* infections (Yang et al., 2022). Both murine lncRNA-CGB Genomic knock-out (KO) models and a human lncRNA-CGB knockdown CD3^+^ T cell model showed impaired ability to control *Mtb* replication and infections (Yang et al., 2022). *B. fragilis*, typically depleted in *Mtb* infections, was found to be a key up-regulator of lnRNA-CGB in mouse models and patients with active TB (Yang et al., 2022) LncRNA-CGB was found to epigenetically regulate IFNγ through interaction with enhancer of zeste homolog 2 (EZH2) (Yang et al., 2022). These findings provide a direct link between microbiota and the immune protection against TB conferred through the gut-lung axis.

A recent study also indicated that parenteral BCG vaccination in murine models causes time-dependent development of gut dysbiosis associated with increased production of butyrate (Jeyanathan et al., 2022). Additionally, BCG induces mild self-limiting, time-dependent intestinal inflammation causing significantly increased intestinal permeability, enabling the leakage of luminal molecules such as butyrate through the epithelium (Jeyanathan et al., 2022). Naïve mice treated with antibiotics and then transplanted with microbiota from BCG-immunized hosts were found to have alveolar macrophages (AM) with elevated MHC II, IL-6, and TNF production at base line and upon stimulation (Jeyanathan et al., 2022). These results indicate that a component of the protection conferred by BCG vaccination occurs through modulation of the lung-gut axis (Jeyanathan et al., 2022).

Conventional anti-TB regimens of isoniazid-rifampicin-pyrazinamide-ethambutol (HRZE) induce significant long-term gut dysbiosis that persist well beyond treatment cessation both in companion murine and human studies (Namasivayam et al., 2017; Wipperman et al., 2017). Subjects showed increased populations of *Erysipelayoclotridium, Prevotella* and *Fusobacterium*, and decreased population of *Blautia*, *Lactobacillus*, *Coprococcus*, *Bifidobacterium* and *Bacteroides genera* compared to latent TB controls (Wipperman et al., 2017). Polysaccharides produced by *Bacteroides* have been demonstrated to induce IL-10 producing Treg cells in mice (Johnson et al., 2018; Erturk-Hasdemir et al., 2019). Similarly, some *Lactobaccillus* spp. also can increase Treg cell populations and contribute to immune tolerance (Ding et al., 2017), *Coprococcus* can modulate IL-1β, IFNγ, and other cytokines in response to fungal stimuli (Schirmer et al., 2016), and *Bifidobacterium* is capable of inducing intestinal Th17 cells in animal models (Tan et al., 2016). This supports the notion that gut dysbiosis drives changes in immune signaling that potentially contribute to the variable efficacy of HRZE treatment (Wipperman et al., 2017). Additionally, the persistence of gut dysbiosis beyond treatment cessation may increase the likelihood of reinfection or other infections in TB patients (Osei Sekyere et al., 2020).

## Probiotics and gut metabolites

Due to the adverse dysbiotic effects, expense and adherence challenge of conventional anti-TB therapy and growing evidence of the role of microbiota in TB, probiotics are now being explored as novel therapeutic avenue (Wipperman et al., 2017; Rahim et al., 2022). Probiotics have been previously shown to be effective in suppressing antibiotic resistant organisms which otherwise require more intensive and expensive treatments (Rahim et al., 2022). *Lacticaseibacillus rhamnosus* isolated from the vagina of healthy women, inhibited *Mtb* growth in culture medium and showed intracellular killing activity against drug sensitive and resistant strains of *Mtb* in a murine macrophage cell line without cytotoxicity (Rahim et al., 2022). A murine experiment involving the intragastric administration of *Lactobacillus casei* indicated a protective role for probiotics against the gut-based adverse reactions of isoniazid and rifampicin through modulation of SCFAs (Li Y. et al., 2022). This was consistent with a larger randomized open label dose–response clinical trial of *Lactobacillus casei* that found reduced anti-TB associated gastrointestinal adverse effects (Lin et al., 2020).

As evidence strongly supports that microbiome immunomodulation is primarily modulated through their secreted metabolites, the use of inactivated microbial cells or their components, termed as postbiotics, is also being investigated (Liu Y. et al., 2021). In a whole-cell screen of a fragment library, a recent study found that the gut microbial metabolite indole propionic acid (IPA) showed significant dose-dependent anti-tubercular *in vitro* potency comparable to first line antibiotics (Negatu et al., 2018). *In vivo* experiments confirmed the effect of IPA through 7-fold reductions in bacterial load in the spleen of *Mtb*-infected mice (Negatu et al., 2018). The antimycobacterial-specific activity *in vitro* of IPA has been shown to occur through mimicking the allosteric inhibitory effect of tryptophan (Trp) on anthranilate synthase TrpE as part of the physiological negative feedback loop on tryptophan synthesis (Kaufmann, 2018; Negatu et al., 2019). Thus, while evidence supports the beneficial role of probiotics and microbial metabolites in TB, it remains limited and should be explored through larger scale animal experiments.

## Conclusion

The existence of the gut-lung axis has been consistently supported through recent literature. Gut microbiota have been shown to influence lung immunity through production of signaling molecules or SCFAs that modulate inflammatory responses in disease states. Significant evidence also supports a role for the gut-lung axis in TB pathogenesis and recent studies have demonstrated direct links between bacterial species and immunity against TB. However, evidence around the changes in microbiota composition in *Mtb* infection and its significance is contradictory and needs to be evaluated in more detail through large scale human studies. The use of antibiotics in TB therapy has been linked to significant gut dysbiosis which may be resolved through supplementation with probiotics. The limited research in the use of probiotics in TB is favorable to its use, but this hypothesis needs to be tested more thoroughly in animal and human studies. The strength of association between TB pathogenesis and microbiota alterations holds promise for the development of new therapeutics particularly through probiotic agents or purified bacterial compounds to be used against TB.

## Author contributions

AE and AK conceived the manuscript. AE wrote the first draft. HS provided editorial and intellectual input and designed the Figure. All authors contributed to manuscript revision and approved the submitted version.

## Funding

AE was supported by an Amuthan Medical Research Grant from James Cook University. AK was supported by an NHMRC Ideas (APP2001262) and Investigator Grant (APP2008715).

## Conflict of interest

The authors declare that the research was conducted in the absence of any commercial or financial relationships that could be construed as a potential conflict of interest.

## Publisher’s note

All claims expressed in this article are solely those of the authors and do not necessarily represent those of their affiliated organizations, or those of the publisher, the editors and the reviewers. Any product that may be evaluated in this article, or claim that may be made by its manufacturer, is not guaranteed or endorsed by the publisher.
